# Overexpression of FRAT1 Is Associated with Malignant Phenotype and Poor Prognosis in Human Gliomas

**DOI:** 10.1155/2015/289750

**Published:** 2015-04-02

**Authors:** Geng Guo, Cheng-liang Zhong, Yang Liu, Xing-gang Mao, Zheng Zhang, Ji Jin, Jing Liu, Liu Yang, Jin-ming Mao, Yu-hong Guo, Yuan-li Zhao

**Affiliations:** ^1^Department of Neurosurgery, Beijing Tiantan Hospital, Capital Medical University, Beijing 100050, China; ^2^Department of Neurosurgery, The First Hospital, Shanxi Medical University, Taiyuan, Shanxi 030001, China; ^3^GCP Center, The First Affiliated Hospital of Tianjin University of Traditional Chinese Medicine, Tianjin 300192, China; ^4^Postdoctoral Programme, China Academy of Chinese Medical Sciences, Beijing 100700, China; ^5^Department of Neurosurgery, The Third Hospital of Mianyang, Mianyang, Sichuan 621000, China; ^6^Department of Neurosurgery, Xijing Hospital, Fourth Military Medical University, Xi'an, Shaanxi 710032, China; ^7^Department of Neurology, The First Affiliated Hospital of Wenzhou Medical University, Wenzhou, Zhejiang 325000, China; ^8^Shanxi Medical University, Taiyuan, Shanxi 030001, China; ^9^Department of Neurology, The First Hospital, Shanxi Medical University, Taiyuan, Shanxi 030001, China

## Abstract

Glioma is the most common malignancy of the central nervous system. Approximately 40 percent of intracranial tumors are diagnosed as gliomas. Difficulties in treatment are associated closely with the malignant phenotype, which is characterized by excessive proliferation, relentless invasion, and angiogenesis. Although the comprehensive treatment level of brain glioma is continuously progressing, the outcome of this malignancy has not been improved drastically. Therefore, the identification of new biomarkers for diagnosis and therapy of this malignancy is of significant scientific and clinical value. FRAT1 is a positive regulator of the Wnt/*β*-catenin signaling pathway and is overexpressed in many human tumors. In the present study, we investigated the expression status of FRAT1 in 68 patients with human gliomas and its correlation with the pathologic grade, proliferation, invasion, angiogenesis, and prognostic significance. These findings suggest that FRAT1 may be an important factor in the tumorigenesis and progression of glioma and could be explored as a potential biomarker for pathological diagnosis, an indicator for prognosis, and a target for biological therapy of malignancy.

## 1. Introduction

Glioma is the most common malignancy of the central nervous system. Approximately 40 percent of intracranial tumors are diagnosed as gliomas [1]. Difficulties in treatment are associated closely with the malignant phenotype, which is characterized by excessive proliferation, relentless invasion, and angiogenesis. Although the comprehensive treatment level of brain glioma is continuously progressing, the outcome of this malignancy has not been improved drastically. For example, patients with the most malignant histopathologic subtype, glioblastoma (GBM), carry the worst prognosis, with a median survival period of less than 12 months, despite having undergone surgical treatment combined with radiotherapy and chemotherapy [2]. Therefore, the identification of new biomarkers for diagnosis and therapy of this malignancy is of significant scientific and clinical value.

The FRAT1 (frequently rearranged in advanced T-cell lymphomas-1) gene, located on human chromosome 10q24.1 [3], encodes a 29 kDa protein comprising 279 amino acids. FRAT1 is a positive regulator of the Wnt/*β*-catenin signaling pathway [4–6] and is overexpressed in many human tumors [7–11]. Recently, we demonstrated that FRAT1 expression is elevated in gliomas [12, 13] and that its expression is correlated with pathologic grade, proliferation, and apoptosis in astrocytomas [13]. The expression of FRAT1 is considered to be crucial for the maintenance of the malignant cellular phenotype. However, little is known about the contribution of FRAT1 expression to the prognosis of glioma. In the present study, we investigated the expression status of FRAT1 in 68 patients with human gliomas and its correlation with the pathologic grade, proliferation, invasion, angiogenesis, and prognostic significance. These results may have important implications in both predicting the clinical prognosis and understanding the biology of these tumors.

## 2. Materials and Methods

### 2.1. Tumor Specimens

Sixty-eight patients with gliomas were included in this retrospective study. All of the cases were surgically treated at the Neurosurgical Department of the Xijing Hospital affiliated to the Fourth Military Medical University from June 2007 to August 2009. The patients included 35 males and 29 females ranging in age from 6 to 76 years (45.6 ± 17.7, mean ± standard deviation (SD)). All tumor tissues were obtained from the initial surgery prior to chemotherapy or radiation therapy. The extents of resection (gross total (greater than 90% resected) or subtotal (60% to 90% resected)) were documented on the basis of the surgeon's intraoperative impression with postoperative radiographic confirmation of the absence or presence of residual disease. The extent of resection was greater than 60% for all patients in the study. Histologic subtypes and pathologic grades of tumors were quantified according to the revised World Health Organization (WHO) criteria for the central nervous system [1] as follows: 10 cases with Grade I, all pilocytic gliomas; 19 cases with Grade II, including 16 diffuse gliomas, 2 oligodendrogliomas, and 1 protoplasmic glioma; 16 cases with Grade III, including 12 anaplastic gliomas and 4 anaplastic oligodendrogliomas; and 23 cases with Grade IV, all glioblastomas. The pathologic grades of all samples were confirmed independently by two pathologists. Five samples of slightly impaired brain tissue fragments from volunteers with cerebral trauma were used as control. Twenty-three patients with glioblastoma were treated with surgery, radiotherapy, and adjuvant chemotherapy. They were followed up every 2 months for at least 24 months posttreatment. This study was approved by the Institutional Review Board of the Xijing Hospital of the Fourth Military Medical University, Xi'an, China. All participants provided written informed consent prior to their participation. For participants who lack mental or physical capacity to consent, written informed consent on behalf of the participant was provided by a legal proxy.

### 2.2. Immunohistochemistry

For immunohistochemistry, 4 *μ*m thick serial sections were cut from paraffin-embedded specimens, mounted on poly-L-lysine-coated slides and incubated overnight at 60°C. The sections were dewaxed in xylene, followed by rehydration with decreasing concentrations of ethanol solutions. For routine pathological examination, deparaffinized sections from all blocks were stained with hematoxylin and eosin. No histological abnormalities were detected in the sections from any of the 5 normal control brain tissues. Heat-induced antigen retrieval was carried out with 0.01 M citrate buffer (pH 6.0) for 10 minutes. Endogenous peroxidase activity and nonspecific binding were blocked with 3% H_2_O_2_ and nonimmune serum, respectively. Sections were then incubated with primary antibodies overnight at 4°C in a humidified chamber. The rabbit anti-human FRAT1 (U-25) polyclonal antibody was used at 1 : 50 dilution (Santa Cruz Biotechnology, Santa Cruz, CA, USA). Mouse anti-human PCNA (PC10) monoclonal antibody, mouse anti-human MMP-9 (2C3) monoclonal antibody, and mouse anti-human CD34 (D-6) monoclonal antibody were diluted 1 : 100 with phosphate buffered saline (PBS) (Santa Cruz Biotechnology, Santa Cruz, CA, USA). The primary antibodies were then detected using the appropriate labeled Streptavidin-Biotin (LSAB) kit (Maixin Biotechnology, Fuzhou, China) according to the manufacturer's instructions. Immunolabeled sections were visualized with 3′,3′-diaminobenzidine tetrahydrochloride (DAB; Sigma, St. Louis, MO, USA) and counterstained with hematoxylin. As a specificity control, PBS was used instead of the primary antibody to exclude nonspecific binding of the secondary antibody. All immunostaining experiments were assessed by an experienced pathologist blinded to all clinical data. Digital microscopic images were captured with the Olympus BX 51 microscope (Olympus, Tokyo, Japan).

### 2.3. Staining Interpretation

The staining results of immunohistochemistry were evaluated by two independent neuropathologists who had no knowledge of the pathologic diagnosis or any clinical data of the tumor specimens. Another independent neuropathologist blinded to the experiment and patients was recruited for disputes in scoring of specific sections. Brown-yellow staining in the cytoplasm was considered positive for FRAT1 and MMP-9; brown-yellow staining in the nucleus was considered positive for PCNA; and brown-yellow staining in vascular endothelial cells was considered positive for CD34. To measure the FRAT1 immunoreactivity score (IRS), proliferative index (Pi), and invasive index (Ii), 10 high-powered (400x) fields (about 1000 cells) were randomly selected for quantification in the most strongly stained tumor area of each section. The FRAT1 immunoreactivity score (FRAT1 IRS) was determined by semiquantitative assessment according to the method described by Friedrich et al. [14]. Values for the percentage of FRAT1-positive tumor cells (0, <1%; 1, 1–25%; 2, 26–50%; 3, 51–75%; 4, >75%) were multiplied by the values for FRAT1 staining intensity (0, no staining; 1, light yellow; 2, buff; 3, brown) to calculate the FRAT1 IRS. Because of the heterogeneous staining intensity of tumor cells, the latter value was determined according to the staining intensity of most cells. Specimens with an IRS score >1 were considered FRAT1 positive. The percentages of PCNA-positive cells and MMP-9-positive cells were regarded as the Pi and Ii of the specimen, respectively. The average values of the Pi and Ii from two neuropathologists were adopted. The microvessel density (MVD) of gliomas was measured according to the method described previously [15]. Briefly, in areas with the most intense neovascularization, individual microvessel counts were made on a 200x magnification field. Any endothelial cell or endothelial cell cluster was considered a single countable microvessel. MVD was expressed as the absolute number of microvessels per 200x field for each case.

### 2.4. Statistical Analysis

All statistical analysis was performed using SAS (Statistical Analysis System), version 9.3 (SAS Institute Inc., Cary, NC, USA). Data were expressed as mean ± SD. Differences in FRAT1 IRS, Pi, Ii, and MVD in different pathologic grades were first analyzed using one-way analysis of variance (ANOVA); then the differences between each of the two groups were further compared by the Student-Newman-Keuls test (SNK test). Differences in the Pi, Ii, and MVD between FRAT1-positive and FRAT1-negative groups were compared using Student's* t*-test. Correlation coefficients of FRAT1 IRS with the Pi, Ii, and MVD were evaluated using Pearson's correlation analysis. The Spearman rank test was used to establish the correlation of histological grades with FRAT1 IRS, Pi, Ii, and MVD. Kaplan-Meier survival analysis was carried out to assess the probability of patient survival, measuring from the time when the diagnosis of GBM was made with subsequent surgical resection to death from any cause. The log-rank test was used to compare the median survival time between the FRAT1-positive and -negative expression groups. Univariate and multivariate analysis were used to define prognostic factors that influenced survival time. Values of *P* < 0.05 were considered statistically significant.

## 3. Results

### 3.1. FRAT1 Is Overexpressed in Glioma

**Figure 1:**
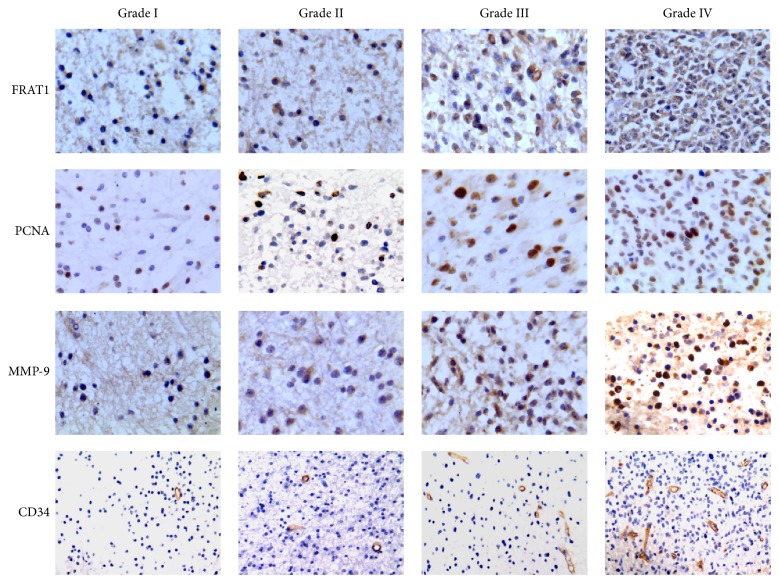
Immunohistochemical analysis of FRAT1 and markers for malignant activity in brain gliomas. Representative images are shown for sections from gliomas with increasing WHO grade (Grades I–IV) that were immunostained for FRAT1, proliferating cell nuclear antigen (PCNA, a marker for proliferation), matrix metalloproteinase-9 (MMP-9, a marker for invasiveness), and CD34 (a microvessel marker). Slides were costained with DAB as chromogen and hematoxylin as counterstain. FRAT1 and MMP-9 immunoreactivity show brown-yellow staining in the cytoplasm of tumor cells; PCNA immunoreactivity shows brown-yellow staining in the nucleus of tumor cells; CD34 immunoreactivity shows brown-yellow staining in vascular endothelial cells. Original magnification ×400 (FRAT1, PCNA, and MMP-9) and ×200 (CD34).

Our previous results suggest that FRAT1 is overexpressed in gliomas as assessed by RT-PCR, western blotting, and immunohistochemistry [12, 13]. To verify these findings for the cohort of 68 glioma patients in this study, we assessed the FRAT1 immunoreactivity of stained sections. Immunopositive tumor cells showed primarily cytoplasmic labeling under light microscopy. The positive expression rate of FRAT1 was 58.82% (40/68), and the mean FRAT1 IRS was 4.25 ± 3.86 for the 68 tumor specimens; however, 5 normal brain tissue specimens had exceedingly weak or absent immunoreactivity for this protein. These results verify our previous findings that FRAT1 is overexpressed in glioma. Representative images of FRAT1 immunostaining are shown in Figure 1, and the related results are given in Table 1.

### 3.2. FRAT1 Is Associated with the Pathologic Grade, Proliferative Index, Invasive Index, and Microvessel Density of Glioma

**Figure 2:**
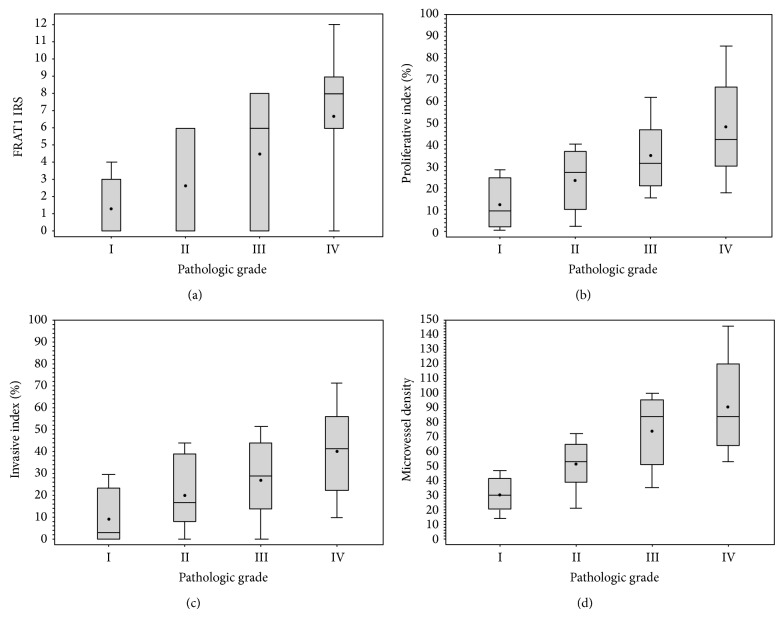
Comparison of the FRAT1 IRS, Pi, Ii, and MVD for glioma specimens of increasing WHO grades. The FRAT1 immunoreactivity score (FRAT1 IRS) (a), the proliferative index (Pi) (b), the invasive index (Ii) (c), and the microvessel density (MVD) (d) were based on the staining results of FRAT1, PCNA, MMP-9, and CD34, respectively. Each of these scores increased significantly with ascending pathologic grade (*P* < 0.05).

We demonstrated previously that FRAT1 expression is associated with pathologic tumor grade and proliferation, as assessed by Ki-67 staining [13]. In this study, the FRAT1 IRS was positively and markedly correlated with increasing WHO grades (*F* = 8.1, *P* = 0.001) (Figure 2(a); Table 1). The cell proliferation marker PCNA was expressed in all tumor specimens (Figure 1), and the Pi of all tumor specimens ranged from 0.8 to 85.3% (33.06 ± 20.93%). With the increasing pathologic grade of glioma, Pi increased markedly (*F* = 13.20, *P* < 0.001) (Figure 2(b); Table 1). The positive expression rate of cell invasion marker MMP-9 was 86.76% (59/68) in tumor specimens (Figure 1). The Ii of tumor specimens ranged from 0.0 to 69.0% (26.70 ± 19.93%). An increase in pathologic grade of glioma was accompanied by a remarkable increase in Ii (*F* = 9.13, *P* < 0.001) (Figure 2(c); Table 1). Microvessels were observed in all tumor specimens (Figure 1). The MVD of brain gliomas ranged from 14 to 145 (66.59 ± 31.05), which increased markedly with the increase in pathologic grade of brain gliomas (*F* = 20.04, *P* < 0.001) (Figure 2(d); Table 1). These results verify our previous findings that FRAT1 expression is associated with the WHO tumor grade and proliferation and extend the results by showing that FRAT1 is also associated with other properties of malignant glioma, including invasiveness and microvessel formation.

**Figure 3:**
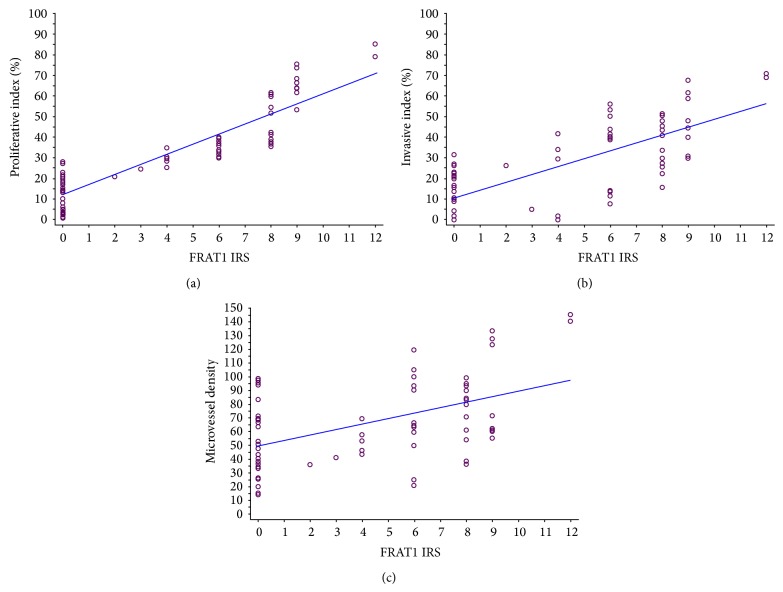
Correlation of the FRAT1 IRS with other malignant activity scores. Scatterplots demonstrate the correlation of the FRAT1 IRS with the Pi, Ii, and MVD in human glioma. A trend line provided in each plot represents the “best fit” as determined by simple linear regression. With increased FRAT1 IRS, the Pi (a), Ii (b), and MVD (c) were increased significantly (*P* < 0.001 for all).

To assess the correlation between the FRAT1 IRS and these other measures of malignancy, we performed Pearson's regression analysis. The Pi (*r* = 0.942, *P* < 0.001), Ii (*r* = 0.731, *P* < 0.001), and MVD (*r* = 0.441, *P* < 0.001) were each positively correlated with the FRAT1 IRS (Figure 3). To confirm these results, we divided the 68 glioma specimens into two groups based on FRAT1 positivity. The Pi, Ii, and MVD were all significantly higher in the FRAT1-positive group than in the FRAT1-negative group (Table 2). Collectively, these results demonstrate that FRAT1 expression may serve as a biomarker for gliomas of different pathological grades and with different malignancy characteristics.

### 3.3. FRAT1 Expression Status Correlates with the Prognosis of GBM

**Figure 4:**
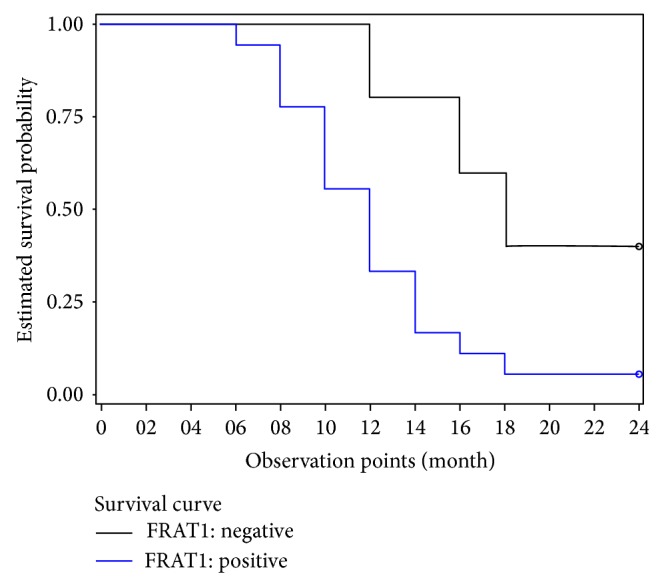
Correlation of FRAT1 positivity with outcome in GBM. Kaplan-Meier survival analysis of GBM patients with FRAT1-positive or -negative expression. FRAT1-positive expression in tumor tissues correlates significantly with inferior survival in comparison with FRAT1-negative expression (*P* = 0.005).

To determine whether FRAT1 expression may have prognostic value, we compared the prognosis of the 23 GBM patients in our study according to the FRAT1 status of the tumor. Patients in the FRAT1-positive group had a lower 2-year overall survival rate (5.56%; 1/18) as compared to FRAT1-negative GBM patients (40%; 2/5). Additionally, the median survival time (12 versus 18 months) was reduced for the FRAT1-positive versus FRAT1-negative groups. Kaplan-Meier survival plots show a statistically significant association between positive FRAT1 expression and poor outcomes among GBM patients (*P* = 0.005; Figure 4).

To determine whether FRAT1 may serve as an independent prognostic factor, we used Cox regression analysis of FRAT1 expression and several other clinicopathological variables on patient survival. Age, FRAT1 positivity, and tumor size were indicated to be important prognostic factors by both univariate Cox regression analysis (Table 3) and multivariate Cox regression analysis (Table 4), whereas the other variables tested (sex, extent of resection, and Karnofsky performance status) did not correlate significantly with overall survival. These results suggest that FRAT1 expression may be indicative of poorer overall survival for GBM patients.

## 4. Discussion

Wnt/*β*-catenin signaling has been reported to be an evolutionarily conserved molecular mechanism in metazoan animals. This pathway plays a critical role in embryogenesis, cell proliferation, differentiation, survival, neural development, and angiogenesis [16–19]. Extensive studies have shown that aberrant activation of the Wnt/*β*-catenin signaling pathway is associated with a broad range of human cancers, including breast cancer, acute leukemia, and colon cancer [7–11, 20–22]. Researchers also have confirmed that the Wnt/*β*-catenin signaling pathway is correlated with the initiation, proliferation, invasion, pathological angiogenesis, and prognosis of glioma [23–28].

FRAT1 was first identified as a protooncogene that contributes to the progression of mouse T-cell lymphomas [29]. With the isolation of GBP, FRAT1's homolog in* Xenopus* [5], FRAT1 was gradually regarded as a potent activator of the Wnt/*β*-catenin pathway. FRAT1 is recruited by Dvl and competes with Axin for the same binding site on GSK-3*β*, leading to the dissociation of GSK-3*β* from a scaffolding complex that contains APC and Axin. The dissociation of GSK-3*β*, in turn, prevents the phosphorylation and consequential degradation of *β*-catenin [6, 30–32]. As a result, unphosphorylated *β*-catenin accumulates in the cytoplasm and translocates to the nucleus. In the nucleus, *β*-catenin combines with T-cell factor (TCF)/lymphoid-enhancing factor (LEF); then they form a transcriptional complex which can increase the expression of oncogenic target genes [33]. This pathway is believed to contribute to tumor progression. Thus, it is reasonable that FRAT1 has been found to be strikingly overexpressed in several human cancers, including esophageal cancer, cervical cancer, breast cancer, ovarian cancer, and non-small-cell lung cancer [7–11].

It is well known that the major characteristics of malignant tumors are unlimited proliferation, invasion, and angiogenesis. In the current study, we confirmed that FRAT1 is generally overexpressed in gliomas and that the expression levels of FRAT1 are significantly positively correlated with increasing WHO grades. We verified that the Pi is increased in gliomas of increasing grade using an alternate marker for proliferation, and we also showed an association of glioma grade with Ii and MVD. Furthermore, we demonstrated that FRAT1 expression is positively correlated with Pi, Ii, and MVD by a variety of statistical techniques. Importantly, overexpression of FRAT1 was shown to correlate with poor overall survival in GBM patients. To the best of our knowledge, this is the first assessment of the contribution of FRAT1 expression to survival of glioma patients.

Collectively, these observations suggest that FRAT1 may play a pivotal role in the development and progression of gliomas due to its multiple biologic activities involved in promoting proliferation, invasion, and angiogenesis. We propose that FRAT1 may be a useful biomarker for molecular diagnosis, an indicator for the prognosis of glioma, and an intriguing candidate target for glioma therapy. Because key genetic, epigenetic, and environmental factors associated with gliomagenesis remain incompletely defined, our findings not only provide more knowledge about the roles that Wnt/*β*-catenin pathway plays during the tumorigenesis of glioma but also contribute to a novel therapeutic strategy for the treatment of patients with glioma. There was a research which reported that the silencing of FRAT1 could increase the phosphorylation of *β*-catenin and lead to a decreased *β*-catenin level [22]. The detailed mechanism underlying the overexpression of FRAT1 in glioma and its exact role in the Wnt/*β*-catenin pathway remain to be further investigated.

## Figures and Tables

**Table 1:** Measurements of FRAT1 IRS, Pi, Ii, and MVD in gliomas.

Glioma tissues	n	FRAT1 IRS	Pi %	Ii %	MVD
Grade I	10	1.30 ± 1.77	12.62 ± 11.24	9.18 ± 12.03	30.36 ± 11.69
Grade II	19	2.63 ± 2.91	23.66 ± 13.69	19.84 ± 15.66	51.08 ± 15.27
Grade III	16	4.50 ± 3.76	35.19 ± 16.08	26.80 ± 18.05	73.81 ± 24.52
Grade IV	23	6.70 ± 3.89	48.23 ± 21.05	39.91 ± 19.12	90.14 ± 29.28

Total	68	4.25 ± 3.86^a^	33.06 ± 20.93^a^	26.70 ± 19.93^a^	66.59 ± 31.05^a^

^a^ *P* < 0.001 as compared among Grades I to IV of brain gliomas (by ANOVA). IRS, immunoreactivity score; Pi, proliferative index; Ii, invasive index; MVD, microvessel density.

**Table 2:** IRS, Pi, Ii, and MVD in FRAT1-positive and FRAT1-negative groups of gliomas.

	All gliomas % (range)	FRAT1-positive %	FRAT1-negative %	*t*	*P*
Pi	33.06 ± 20.93 (0.8 to 85.3)	45.59 ± 16.78%	14.03 ± 8.51%	*t* = 10.21	*P* < 0.001
Ii	26.70 ± 19.3 (0 to 69.0)	36.72 ± 18.29%	11.49 ± 10.51%	*t* = 7.21	*P* < 0.001
MVD	66.59 ± 31.05 (14 to 145)	75.10 ± 31.58	53.68 ± 25.75	*t* = 2.94	*P* = 0.0045

Pi, proliferative index; Ii, invasive index; MVD, microvessel density.

**Table 3:** Univariate Cox regression analysis of possible contribution to survival^a^.

Variable		*n*	Hazard ratio (95% CI)	*P*
Age	≥50 y	15	8.928 (2.363–33.734)	0.0012
	<50 y	8		

Sex	Female	10	1.485 (0.609–3.625)	0.3848
	Male	13		

Extent of resection	Total	14	0.428 (0.169–1.086)	0.0741
	Subtotal	9		

Karnofsky performance status	≥80	17	0.741 (0.281–1.958)	0.5458
	<80	6		

FRAT1 positivity	+	18	4.817 (1.307–17.751)	0.0182
	−	5		

Tumor size	≥4 cm	16	3.974 (1.298–12.165)	0.0157
	<4 cm	7		

^a^CI: confidence interval.

**Table 4:** Multivariate Cox regression analysis of possible contribution to survival^a^.

Variable	Hazard ratio (95% CI)	*P*
Age	14.18 (1.461–137.550)	0.0222
FRAT1 positivity	13.97 (1.157–168.681)	0.0380
Tumor size	37.43 (2.987–469.005)	0.0050

^a^CI: confidence interval.
